# The Impact of COVID-19 on the Monitoring of Pregnancy and Delivery of Pregnant Women in the Dominican Republic

**DOI:** 10.3390/healthcare10112266

**Published:** 2022-11-11

**Authors:** Mar Requena-Mullor, Jessica García-González, Ruqiong Wei, Raúl Romero-del Rey, Raquel Alarcón-Rodríguez

**Affiliations:** 1 Department of Nursing, Physiotherapy and Medicine, Faculty of Health Sciences, University of Almería, 04120 Almería, Spain; 2 Department of Rehabilitation Medicine, The First Affilited Hospital of Guangxi Medical University, Nanning 530021, China

**Keywords:** childbirth, coronavirus, perinatal care, pregnancy outcomes

## Abstract

Pregnancy monitoring is vital to guaranteeing that both the foetus and the mother are in optimal health conditions. WHO protocols recommend at least eight medical examinations during the pregnancy period. While the cancellation or reduction of appointments during pregnancy due to the pandemic may help reduce the risk of infection, it could also negatively influence perinatal outcomes and the birthing process. The aim of this research was to analyse the differences in perinatal outcomes and birth characteristics in two groups of pregnant women: women who gave birth before and during the pandemic, and whether these differences are due to changes in pregnancy monitoring because of the COVID-19 situation. A retrospective study was carried out from July 2018 to December 2021, at the Santo Domingo Hospital (Dominican Republic). A total of 1109 primiparous pregnant women were recruited for this study during the birthing process and perinatal visits. The results describe how women who gave birth before the pandemic had greater control and monitoring of their pregnancy, more doctor visits (*p* = 0.001), fewer caesarean sections (*p* = 0.006), and more skin-to-skin contact after birth (*p* = 0.02). During the COVID-19 pandemic, pregnant women’s attendance at routine pregnancy monitoring, both doctor visits and ultrasound scans, has decreased, leading to an increase in the number of caesarean and instrumental deliveries. At the perinatal level, processes such as skin-to-skin contact after birth between mother and newborn or the introduction of early breastfeeding in the delivery room have also been reduced.

## 1. Introduction

Coronavirus disease 2019 (COVID-19) is an emerging infection caused by the coronavirus (SARS-CoV-2) and transmitted mainly by droplets. This novel coronavirus is highly contagious and has spread rapidly around the world in just a few months [1]. As it is considered a global infection, rapid advances are being made in both the understanding of the disease´s mechanisms of action and possible treatments. However, although several guidelines and protocols for pregnancy care have been published, most of them focus on the care of COVID-19-positive pregnant women, with few official documents monitoring and educating uninfected pregnant women [2].

The World Health Organization (WHO) states that prenatal care could reduce maternal and perinatal mortality and morbidity directly by detecting problems that may arise in pregnancy and treating them, and indirectly, by detecting women who may suffer from complications in the future and referring them to an appropriate specialist [3]. During the pandemic, hospitals have had to adapt their protocols to this unique situation, and have had to drastically change how healthcare is provided, even suspending it in some cases [4]. In Maternity and Children’s Hospitals, visitors and accompaniment have been restricted, replacing some visits with telematic consultations [5,6]. These changes in care, coupled with concerns about the possibility of infection to the mother and baby [7], lead women to face the dilemma of whether or not they should go to the hospital for their monitoring visits. Many pregnant women feel that their questions are irrelevant in the current health context of COVID-19 and that their problems are not serious enough to warrant a hospital visit, in order to not overburden the health situation, already overburdened by infected COVID-19 patients [8].

Currently, the WHO recommends at least eight monitoring examinations during pregnancy. Cancelling appointments or not going to the hospital may reduce the risk of contracting the virus, but it may also have a negative effect on pregnancy outcomes [3]. Therefore, studying the impact of the changes that are taking place is so necessary, since the impact of this disease on pregnant women is stronger, as they are more vulnerable during this time, hence, the importance of conducting our study, in order to establish action protocols to avoid these possible differences before and after the pandemic. Changes in the maternal immune system and cardiopulmonary system occur during pregnancy, so it can be expected that they are more susceptible to developing COVID-19 with more severe complications [9]. There is some evidence that the clinical symptoms and outcomes of COVID-19 in pregnant women are similar to those of other adults, and the likelihood of transmission to the baby during childbirth or breastfeeding is low [10,11]. However, an increased risk of associated complications in the second half of pregnancy [12,13,14], preterm childbirth, and hospital admission [15,16] has been found as a result of the disease. In addition, COVID-19 could have similar effects as other viruses such as the H1N1 influenza virus or SARS and MERS viruses, which cause complications in pregnant women such as increased ICU admission, intubation, renal failure, or death during gestation [17,18]. Previous studies indicate that, during the pandemic, women felt that the pre and postnatal care received was inadequate, and they were emotionally distressed, isolated, and lacked necessary support [19,20,21,22]. This fact could be very concerning because emotional distress during pregnancy can lead to premature childbirth, low birth weight, postpartum depression, and infant developmental delays [23,24].

Despite the evidence found on the emotional distress experienced by pregnant women during the COVID-19 pandemic [20,21,22], more studies are needed to analyse the impact of the changes taking place in the monitoring and control of pregnancy and their effects on perinatal and birth outcomes. Therefore, this study aimed to analyse whether there are differences in perinatal outcomes and childbirth characteristics between women who gave birth before the pandemic and those who gave birth during the pandemic; and whether these differences were due to changes in pregnancy monitoring resulting from the current COVID-19 situation.

## 2. Materials and Methods

### 2.1. Design

A retrospective study was conducted on primigravid women who gave birth at the Santo Domingo Hospital (Dominican Republic). This study aimed to analyse how the control and monitoring of the pregnancy before and during the pandemic influenced delivery processes and perinatal outcomes.

### 2.2. Study Population and Data Collection

A total of 1109 primiparous pregnant women who gave birth from July 2018 to December 2021 were enrolled in this study. Participants were recruited using the Hospital´s Electronic Book of Deliveries, which lists all childbirths and their characteristics. The study included all primigravid pregnant women with a singleton gestation who delivered during the study period. These women had to have a clinical history in the hospital and all data about their delivery had to be recorded in the Electronic Book of Deliveries. The inclusion criteria in this study were: (1) full-term pregnancy (≥37 weeks’ gestation) and low-risk pregnancy, (2) nulliparity with a singleton pregnancy with no known fetal abnormality (3) no use of assisted reproductive technology and (4) no perinatal complications. Exclusion criteria were: (1) chronic conditions prior to pregnancy, (2) multiple pregnancies, (3) gestational diabetes or preeclampsia, (4) mental health, cognitive and psychiatric problems, and (5) women with COVID-19 infection.

Pregnant women who decided to participate in this study were divided into groups. The first one (*n* = 496) included women who had given birth before the pandemic, from July 2018 to March 2020. The other (*n* = 613) was formed by pregnant women who had given birth after the pandemic was declared, from March 2020 to December 2021. Data related to pregnancy monitoring, delivery processes, and perinatal outcomes were collected from participants’ medical records by a single midwife using the Hospital´s Electronic Book of Deliveries. This Book of Deliveries is a clinical information system with seven modules, one of which is the “maternal-child”. The information collected was grouped into socio-demographic variables (age, nationality and consumption of tobacco and alcohol), pregnancy control (number of visits during gestation, weeks of gestation at first visit, first/second/third-trimester ultrasound, maternal education, delivery room visit and diabetes screening), delivery variables (type of beginning and ending of childbirth, accompaniment, position, anaesthesia) and perinatal variables (child weight, Apgar score at first, fifth and tenth minute, pH of the umbilical cord artery (UA), pH of the umbilical cord vein (UV), skin-to-skin contact after birth, early breastfeeding and type of resuscitation).

### 2.3. Ethical Considerations

The research study was approved by the Ethics Committee of the Institute of Human Sexuality of the School of Medicine of the Autonomous University of Santo Domingo (Protocol number: CEI-ISH-001-2021). All the procedures were performed following the ethical standards of the Helsinki Declaration. Participation in this study was voluntary. Pregnant women who volunteered to participate in the study signed an informed consent form after being informed about the objectives of the study and their right to withdraw from the study at any time. The absolute anonymity and confidentiality of the data provided were guaranteed through the generation of a personal code.

### 2.4. Data Analysis

After creating a database with the collected information, a descriptive analysis of the continuous variables was carried out using means and standard deviations, whereas absolute and relative frequency distributions were calculated for categorical variables.

In the bivariate analysis, for the comparison of qualitative variables, the Chi-square test (χ^2^) was applied, with a value of *p* < 0.05 considered significant. For the comparison of means of quantitative variables, the Mann-Whitney U test was used after confirmation through the normality test (Kolmogorov-Smirnov test) that none of the study variables followed a normal distribution. The level of statistical significance was established for a value of *p* < 0.05. The SPSS statistical software package (SPSS 25.0 for Windows) was used for all the statistical analyses.

## 3. Results

A population of 1109 pregnant women participated in this study on pregnancy monitoring and perinatal outcomes in the pre-COVID-19 era and during the pandemic. The mean age of the participants was 27.5 ± 6.22 years, and the mean gestational age at delivery was 39.12 ± 1.82 weeks. The origin of pregnant women was 81.5% Dominican, while the rest were Haitian (18.5%). A total of 13.1% of pregnant women declared to have toxic habits such as alcohol or tobacco. Of the total number of childbirths, only 47.4% of the newborn babies were female and 52.6% male, with a mean weight of 3.22 ± 0.49 kg.

The differences in mean gestational age at which women attended their first visit were not statistically significant (Table 1). In fact, before the pandemic, the mean gestational age was 10.98 ± 6.91 weeks, while during the pandemic, it was 11.98 ± 7.41 weeks. The number of hospital visits for routine pregnancy monitoring was lower during the pandemic (8.78 ± 2.8) than before the pandemic (9.77 ± 2.5). There are also significant differences in the number of women who attended their first ultrasound appointment. Results show that 91% of women attended this appointment before the COVID-19 pandemic, while only 86.8% of women attended it during the pandemic. Attendance of maternal education courses decreased during the pandemic (14.5% before the COVID-19 pandemic versus 3.8% during the COVID-19 pandemic; *p* < 0.001). The number of visits to the delivery room also decreased significantly during the pandemic (7.3% before the COVID-19 pandemic versus 1.6% during the COVID-19 pandemic; *p* < 0.001).

Table 2 shows data related to the delivery processes for women giving birth before and during the pandemic. The number of instrumental and caesarean deliveries during the pandemic increased significantly compared to before the pandemic. Instrumental delivery increased from 16.1% before the pandemic to 19.7% during the pandemic and caesarean from 17.9% to 23.7%. The main kind of anaesthesia used before and during the pandemic was the epidural technique; although, during the pandemic period, the use of nitrous oxide as an anaesthetic technique suffered a significant decrease (*p* = 0.01).

Before the COVID-19 pandemic, epidural anaesthesia was mainly used for lithotomies (43.5%), nitrous oxide anaesthesia was mostly used for the plantar position (59.1%) and spinal anaesthesia for all supine decubitus positions (100%). During the COVID-19 pandemic, a decrease in epidural anaesthesia was observed for lithotomies (35.9%), with increased use for the plantar position (44.3%). There was also a decrease in the use of nitrous oxide anaesthesia for all positions in the expulsive period. Spinal anaesthesia continued to be used mainly for the supine decubitus position (98.6%).

Regarding obstetric procedures, before the COVID-19 pandemic, epidural anaesthesia and nitrous oxide were mostly used for vaginal deliveries (67.9% and 77.3%, respectively). Spinal anaesthesia was used in 100% of cesarean sections. During the COVID-19 pandemic, there was a decrease in the use of epidurals for vaginal deliveries and a slight increase in instrumental deliveries. The use of nitrous oxide also declined for vaginal deliveries and was no longer used for instrumental deliveries. Spinal anaesthesia continued to be used mainly for cesarean deliveries. The detailed results are presented in Table 3.

The number of women who performed skin-to-skin contact with their newborns decreased during the pandemic (82.3% before versus 76.8% during the pandemic). The process of early breastfeeding of newborns also decreased during the pandemic (76.0% before versus 70.6% during the pandemic). These differences were statistically significant in both cases (Table 4). The other perinatal variables such as birth weight, Apgar, UA pH, UV pH, or cardiopulmonary resuscitation showed no statistically significant differences when compared between the two groups of pregnant women.

Finally, when comparing the birth process and perinatal outcome variables with the number of visits during gestation, maternal education attendance, and whether the first ultrasound was performed, no statistically significant differences were found.

## 4. Discussion

Data from this study shows that women who gave birth during the pandemic had less follow-up pregnancy monitoring, missed scheduled appointments such as ultrasound examinations, and had an increased number of instrumental and caesarean deliveries.

### 4.1. The Importance of Pregnancy Monitoring: Routine Visits and Ultrasound Scans

The new WHO antenatal care mode indicates that the number of contacts a pregnant woman should have with health professionals throughout her pregnancy should be increased from four to eight. According to the WHO, prenatal care with at least eight visits could reduce perinatal deaths by up to 8 per 1000 births [3]. This update was due to the results of numerous studies, among which the WHO itself highlights a systematic review consisting of seven clinical trials: four conducted in high-income countries and three in low- and middle-income countries. In these articles, women were divided into two groups: the first group consisted of pregnant women who followed a standard follow-up protocol, and the other group consisted of pregnant women who had a follow-up with a reduced number of visits varying from 4 to 5. As a result of this systematic review, the reduced visit group showed a significant increase in perinatal mortality [25]. Although the evidence indicates the importance of routine visits, pregnant women in this study who gave birth during the pandemic had a lower mean number of visits because of the COVID-19 pandemic. This event has not only happened in our study, but is a phenomenon that has happened all over the world: in England, in hospitals such as St. George’s, pregnant women went to the emergency room less often [26]. Authors attribute this to the fact that women possibly thought that going to the hospital was avoidable except for at the time of delivery. In this regard, the Indian Department of Obstetrics and Gynaecology published that, during the pandemic, 32% of women did not comply with the recommended number of visits [27]. This event was attributed to pregnant women’s fear of infection and the fact that many of them considered the process of pregnancy to be physiological and were safer at home. In China, approximately 50% of women who completed a national survey had considered cancelling or cancelled their monitoring due to fear of infection [18]; while other countries, such as Israel and the United States, also observed a decrease in attendance and an increase in cancellations for the same reasons [28,29,30].

Nevertheless, failure to perform the first ultrasound has not been associated with adverse outcomes, although other studies have associated it with a higher rate of induction of labour due to prolonged pregnancy, as there may be errors in the dating of the week of gestation [31,32]. However, a systematic review including 11 clinical trials and involving 37,505 pregnant women has been found in line with our results. This study concludes that early ultrasounds do not affect rates of caesarean section, perinatal mortality or newborn birth weight [33]. This could be because, even if pregnant women do not attend the first ultrasound scan, the two subsequent ones are performed, in which different anomalies may also be detected early on in the pregnancy, and the necessary measures are taken.

### 4.2. Maternal Education Cancellation Repercussions

Face-to-face maternal education classes were suspended during the pandemic, so attendance was not possible. Therefore, in this study, attendance or non-attendance of these classes could not be related to changes in the birth process and perinatal data. These findings are consistent with other studies whose results indicate that type of delivery, Apgar, neonatal resuscitation and breastfeeding are not influenced by attendance or non-attendance of these classes, although some relationship has been found with prematurity [34,35,36,37]. There is some difficulty in comparing studies that analyse the effectiveness of maternal education since the benefits of maternal education are based on good group dynamics, content, and interventions, which tend to be different in the case groups of each study.

### 4.3. The Childbirth Process in Parturient Women before and during the COVID-19 Pandemic

The results indicate that women in both groups have similar percentages of planned caesarean sections. However, there is an increase in unscheduled caesarean sections (almost 6%) in women who gave birth during the pandemic. This may be due to an increase in complications in women who gave birth during the pandemic, which some authors attribute to higher cortisol levels triggered by the stress compounded by the COVID-19 situation [29], although it could be a chance finding that needs further research. In contrast, Krawczyk et al. [38] observed a decrease in the number of cesarean sections during the pandemic compared to previous years.

In addition, an increase in the percentage of instrumental deliveries has been observed during the pandemic. Authors such as Justman et al. [29] argue that this increase is not due to the pandemic, but rather, to risk factors specific to the woman or the situation that increases in the second stage of labour: epidural, foetal macrosomia, the newborn’s head position, nulliparity [29,37,39,40]. Other studies have not found differences in vaginal, caesarean, and instrumental delivery rates [39,41,42].

Although the findings are not significant in terms of accompaniment during childbirth, there are differences between the two groups. During the pandemic, more women had to give birth alone. The different types of anaesthesia used were similar in the two groups. However, the use of nitrous oxide decreased during the pandemic, through its withdrawal from delivery rooms at the beginning of the pandemic because of possible aerosol production. This withdrawal was endorsed by the Society of Fetal Medicine and the Society of Obstetrics, Analgesia, and Neonatology in the United States [43].

Regarding the use of anaesthesia during the COVID-19 pandemic, there was a decrease in the use of epidurals for vaginal deliveries and a slight increase in instrumental deliveries. Spinal anaesthesia continued to be used mainly for cesarean deliveries, with results consistent with other works, in which spinal anaesthesia was performed in 80% of cesarean deliveries [38].

### 4.4. Perinatal Data of Newborns before and during the COVID-19 Pandemic

Pregnant women showed similar means and percentages of variables associated with perinatal outcomes for both groups (before and during the COVID-19 pandemic). Several authors have also analysed pH, Apgar, birth weight and resuscitation values in women who gave birth before and during the pandemic and have not found significant differences in perinatal outcomes [29,39,41]. In contrast, other studies conclude that women who have given birth during the pandemic have had lower rates of preterm births [25,40], although this is not reflected in our study. Although not all the causes that lead to this situation are yet defined, further research must be carried out on this issue, which seems to be related to home confinement.

Currently, most evidence indicates that mothers should breastfeed their infants regardless of COVID-19 infection, arguing that transmission of the virus by this route is unlikely and that the benefits outweigh the risks [44]. However, some isolated studies advise infected mothers not to breastfeed or, if any relative is infected or suspected to be infected, not to offer to breastfeed their newborns [45,46,47]. Therefore, it seems reasonable that breastfeeding rates have declined during the pandemic [19].

At the beginning of the pandemic, skin-to-skin contact recommendations were controversial. Some authors recommended early clamping and separation of the newborn from the mother until 14 days after birth [18,45]. According to these early studies, strict protocols were followed in many maternity hospitals, as they were in the maternity hospital where this study was conducted. Our study shows that, during the pandemic, the percentage of skin-to-skin contact decreased. However, more recent studies by the WHO and paediatric societies recommend skin-to-skin contact and breastfeeding unless mothers have a very severe infection that prevents it [6,10].

### 4.5. Limitations and Strengths of This Study

One main challenge of this study was understanding the factors that could have influenced the decrease in the number of pregnancy monitoring visits. While fear of infection has had a strong influence on women’s pregnancies, there could be other factors unrelated to the virus that have played a role as well. These include some political measures that could have made attendance difficult, such as arranging childcare at home when schools are closed, the inability to ask for help from relatives or friends, transport difficulties, etc.

However, the data obtained shed light on the situation and could provide insight into whether the measures carried out were the right ones or whether other alternatives were necessary. In addition, the study could contribute to early decision-making in future situations in which attendance at routine visits is again disrupted. This study could also guide other researchers to look for positive effects of routine thorough pregnancy management on birth and perinatal outcomes.

## 5. Conclusions

During the COVID-19 pandemic, pregnant women´s attendance at routine pregnancy monitoring, both doctor visits and ultrasound scans, has decreased, leading to an increase in the number of caesarean and instrumental deliveries. At the perinatal level, processes such as skin-to-skin contact after birth between mother and newborn or the introduction of early breastfeeding in the delivery room have also been reduced. All maternity services should guarantee quality pregnancy monitoring and care for women during pregnancy and the puerperium in unique or difficult situations; thus, protocols and clinical practice guidelines that ensure safe face-to-face access for pregnant women and their partners must be developed.

## Figures and Tables

**Table 1:** Comparison of pregnancy control data between the two groups, “before the COVID-19 pandemic” and “during the COVID-19 pandemic”.

Variables		Pregnancies before the COVID-19 Pandemic (*n* = 496)	Pregnancies during the COVID-19 Pandemic (*n* = 613)	*p*-Value
Gestation week first visit ^1^		10.98 ± 6.91	11.61 ± 7.41	0.14 *
Visit number ^1^		9.77 ± 2.5	8.78 ± 2.88	0.001 *
First trimester ultrasound	No	44 (9%)	80 (13.2%)	0.03 **
	Yes	446 (91%)	528 (86.8%)	
Second trimester ultrasound	No	19 (3.9%)	39 (6.4%)	0.06 **
	Yes	471 (96.1%)	569 (93.6%)	
Third trimester ultrasound	No	13 (2.7%)	23 (3.8%)	0.29 **
	Yes	476 (97.3%)	585 (96.2%)	
Maternal education	No	424 (85.5%)	573 (93.5%)	<0.001 **
	Yes	72 (14.5%)	40 (6.5%)	
Visit to delivery room	No	460 (92.7%)	603 (98.4%)	<0.001 **
	Yes	36 (7.3%)	10 (1.6%)	
Screening GD	No	40(8.1%)	53 (8.75%)	0.72 **
	Yes	455 (91.9%)	558 (91.3%)	

^1^ Values are expressed as mean ± SD. *p*-value obtained using * Mann–Whitney U test for continuous variables or ** Chi-squared test for categorical variables. GD = gestational diabetes.

**Table 2:** Comparison of the data on the labour process between the two groups, “before the COVID-19 pandemic” and “during the COVID-19 pandemic”.

Variables		Deliveries before the COVID-19 Pandemic (*n* = 496)	Deliveries during the COVID-19 Pandemic (*n* = 613)	*p*-Value
Gestation week delivery ^1^		39.14 ± 1.89	39.11 ± 1.77	0.79 *
Position in the expulsion	Plantar position	205 (41.3%)	267 (43.6%)	0.64 **
	Lithotomy	185 (37.3%)	181 (29.5%)	
	Supine Cubitus	89 (17.9%)	146 (23.8%)	
	Others	17 (3.4%)	19 (3.2%)	
Accompaniment	None	20 (4%)	37 (6%)	0.38 **
	Partner	420 (84.7%)	498 (81.2%)	
	Mother	33 (6.7%)	47 (7.7%)	
	Others	23 (4.65%)	31 (5.1%)	
Anaesthesia	Epidural	340 (68.5%)	409 (66.7%)	0.01 **
	Spinal	47 (9.5%)	72 (11.7%)	
	Nitrous oxide	22 (4.4%)	3 (0.5%)	
	No	87 (17.5%)	129 (21%)	
Onset of labour	Spontaneous	301 (60.7%)	383 (62.5%)	0.79 **
	Induced	178 (35.9%)	208 (33.9%)	
	Cesarean	17 (3.4%)	22 (3.6%)	
Completion of delivery birth	Vaginal	327 (65.9%)	347 (56.6%)	0.006 **
	Instrumental	80 (16.1%)	121 (19.7%)	
	Cesarean	89 (17.9%)	145 (23.7%)	

^1^ Values are expressed as mean ± SD. *p*-value obtained using * Mann–Whitney U test for continuous variables or ** Chi-squared test for categorical variables.

**Table 3:** Type of anaesthesia for position in the expulsion and obstetric procedures “before the COVID-19 pandemic” and “during the COVID-19 pandemic”.

Deliveries before the COVID-19 Pandemic						*p*-Value
Type of Anesthesia		Epidural	Spinal	Nitrous Oxide	No Anesthesia	
Position in the expulsion	Plantar position	140 (41.2%)	-	13 (59.1%)	52 (59.8%)	0.001 *
	Lithotomy	148 (43.5%)	-	6 (27.3%)	31 (35.6%)	
	Supine Cubitus	41 (12.1%)	47 (100%)	1 (4.5%)	-	
	Others	11 (3.2%)	-	2 (9.1%)	4 (4.6%)	
Completion of delivery birth	Vaginal	231 (67.9%)	-	17 (77.3%)	79 (90.8%)	0.001 *
	Instrumental	68 (20%)	-	4 (18.2%)	8 (9.2%)	
	Cesarean	41 (12.1%)	47 (100%)	1 (4.5%)	-	
**Deliveries during the COVID-19 Pandemic**						***p*** **-Value**
**Type of Anesthesia**		**Epidural**	**Spinal**	**Nitrous Oxide**	**No Anesthesia**	
Position in the expulsion	Plantar position	181 (44.3%)	-	1 (33.3%)	85 (65.9%)	0.001 *
	Lithotomy	147 (35.9%)	1 (1.4%)	-	33 (25.6%)	
	Supine Cubitus	72 (17.6%)	71 (98.6%)	2 (66.7%)	1 (0.8%)	
	Others	9 (2.2%)	-	-	10 (7.8%)	
Completion of delivery birth	Vaginal	236 (57.7%)	1 (1.4%)	1 (33.3%)	109 (84.5%)	0.001 *
	Instrumental	101 (24.7%)	1 (1.4%)	-	19 (14.7%)	
	Cesarean	72 (17.6%)	70 (97.2%)	2 (66.7%)	1 (0.8%)	

*p*-value obtained using * Chi-squared test for categorical variables.

**Table 4:** Comparison of perinatal data between the two groups, “before the COVID-19 pandemic” and “during the COVID-19 pandemic”.

Variables		Newborns before the COVID-19 Pandemic (*n* = 496)	Newborns during the COVID-19 Pandemic (*n* = 613)	*p*-Value
Newborn weight, in kg ^1^		3.215 ± 465	3.231 ± 510	0.58 *
Apgar score 1 min ^1^		8.77 ± 1.41	8.84 ± 1.14	0.37 *
Apgar score 5 min ^1^		9.71 ± 1.07	9.79 ± 0.80	0.14 *
Apgar score 10 min ^1^		9.86 ± 0.95	9.90 ± 0.67	0.35 *
pH UV ^1^		7.26 ± 0.35	7.27 ± 0.07	0.28 *
pH UA ^1^		7.11 ± 0.79	7.18 ± 0.46	0.12 *
Skin to skin	No	88 (17.7%)	142 (23.2%)	0.02 **
	Yes	408 (82.3%)	471 (76.8%)	
Delayed umbilical cord clamping	No	202 (40.7%)	282(46%)	0.07 **
	Yes	294 (59.3%)	331 (54%)	
Cardiopulmonary resuscitation	No	423 (85.3%)	532 (86.8%)	0.47 **
	Yes	73 (14.7%)	81 (13.2%)	
Breastfeeding in delivery room	No	119 (24%)	180 (29.4%)	0.04 **
	Yes	377 (76%)	433(70.6%)	

^1^ Values are expressed as mean ± SD. *p*-value obtained using * Mann–Whitney U test for continuous variables or ** Chi-squared test for categorical variables.

## Data Availability

Data of this study are stored in an SPSS software Project.
